# Comparison of Lives Saved Tool model child mortality estimates against measured data from vector control studies in sub-Saharan Africa

**DOI:** 10.1186/1471-2458-11-S3-S34

**Published:** 2011-04-13

**Authors:** David A Larsen, Ingrid K Friberg, Thomas P Eisele

**Affiliations:** 1 Department of International Health and Development, Tulane University School of Public Health and Tropical Medicine, New Orleans, LA, USA; 2 Department of International Health, Johns Hopkins Bloomberg School of Public Health, Baltimore, MD, USA

## Abstract

**Background:**

Insecticide-treated mosquito nets (ITNs) and indoor-residual spraying have been scaled-up across sub-Saharan Africa as part of international efforts to control malaria. These interventions have the potential to significantly impact child survival. The Lives Saved Tool (LiST) was developed to provide national and regional estimates of cause-specific mortality based on the extent of intervention coverage scale-up. We compared the percent reduction in all-cause child mortality estimated by LiST against measured reductions in all-cause child mortality from studies assessing the impact of vector control interventions in Africa.

**Methods:**

We performed a literature search for appropriate studies and compared reductions in all-cause child mortality estimated by LiST to 4 studies that estimated changes in all-cause child mortality following the scale-up of vector control interventions. The following key parameters measured by each study were applied to available country projections: baseline all-cause child mortality rate, proportion of mortality due to malaria, and population coverage of vector control interventions at baseline and follow-up years.

**Results:**

The percent reduction in all-cause child mortality estimated by the LiST model fell within the confidence intervals around the measured mortality reductions for all 4 studies. Two of the LiST estimates overestimated the mortality reductions by 6.1 and 4.2 percentage points (33% and 35% relative to the measured estimates), while two underestimated the mortality reductions by 4.7 and 6.2 percentage points (22% and 25% relative to the measured estimates).

**Conclusions:**

The LiST model did not systematically under- or overestimate the impact of ITNs on all-cause child mortality. These results show the LiST model to perform reasonably well at estimating the effect of vector control scale-up on child mortality when compared against measured data from studies across a range of malaria transmission settings. The LiST model appears to be a useful tool in estimating the potential mortality reduction achieved from scaling-up malaria control interventions.

## Background

Malaria was estimated to have directly caused over 715,000 child deaths in 2008 in sub-Saharan Africa [1]. Its indirect influence on mortality is likely even higher [2,3]. Fortunately, vector control interventions, such as insecticide treated mosquito nets (ITNs) and indoor residual spraying (IRS), have been shown to be highly effective in preventing malaria morbidity and mortality among children in malaria endemic settings [4,5]. These interventions have been scaled-up across sub-Saharan Africa as part of international efforts to control malaria and have the potential to significantly impact child mortality.

Unfortunately, vital registration data to measure changes in child mortality are not available across most sub-Saharan African countries. While birth histories within national surveys are useful for obtaining trends in all-cause child mortality at the national level, they do not typically measure cause of death using a linked postmortem verbal autopsy. Most demographic surveillance system sites lack sufficient external validity to estimate child mortality rates or causes at the national level. The Lives Saved Tool (LiST), a part of the Spectrum policy modeling package, was developed to provide national or regional estimates of cause-specific mortality based on the extent of intervention coverage scale-up. Several interventions specific to malaria can be modeled with LiST, including vector control (ITNs and IRS), intermittent preventive treatment to prevent malaria in pregnancy (IPTp), and appropriate malaria case management. LiST can be used to estimate historic changes in child mortality in countries where vital registration data are not available or to estimate the potential impact of future programs that affect child mortality.

While mortality reductions estimated by LiST have performed well when compared against measured data following the scale-up of packages of child survival interventions in various settings [6-8], the model has not been compared specifically to studies that measured changes in child survival following the scale-up of vector control interventions for preventing *Plasmodium falciparum* malaria. Here we compare the percent reduction in all-cause child mortality estimated by LiST against measured reductions in all-cause child mortality from four vector control studies in sub Saharan Africa.

## Methods

We compared reductions in all-cause child mortality estimated by LiST to studies that assessed reductions in all-cause child mortality following the scale-up of vector control interventions. In order to compare the reduction in mortality estimated by LiST to the measured results from studies, the following data had to be available for input into the model: baseline all-cause child mortality rate, the proportion of all-cause child deaths due to malaria, and yearly population coverage of vector control from baseline through the end of the study. Also needed was an all-cause child mortality rate at follow-up to vector control scale-up, or the rate in a contemporaneous control group for comparison to the modeled results. An additional criterion for the purposes of this evaluation was that the study must have had vector control intervention scale-up done in the absence of the scaling-up of other child survival interventions.

We searched PubMed for studies published since 1990 in Africa that met these criteria, using the terms malaria, vector control, and child mortality. Four studies were identified for comparison with List [9-12] (Table 1). Of these, 2 were community randomized controlled trials assessing the impact of vector control on all-cause child mortality, with one assessing the impact of ITNs in rural Gambia [9] and the other insecticide-treated curtains (ITCs) in rural Burkina Faso [10]. Another study measured the effect of social marketing of ITNs on all-cause child mortality in rural Tanzania [11]. The final study included was a longitudinal observational study measuring the association of ITN use and all-cause child mortality under program conditions in 4 areas of rural Kenya [12]. The four studies were in rural areas, representing both east and west Africa, and covered a range of malaria transmission intensities.

**Table 1:** Characteristics of Studies included in validation analysis

Country	Study Area	Years	Study Design	Intervention	Relative Risk cited by the studies (95% CI)
The Gambia [9]	The Gambia	1991-92	Community randomized control trial	ITNs	0.95^a^ (0.71 – 1.28) 0.55^b^ (0.30 – 1.01)
Burkina Faso [10]	Oubritenga	1994-96	Community randomized control trial	Insecticide treated curtains	0.85^c^ (0.70 – 1.04)
Tanzania [11]	Kilombero and Ulanga	1997-99	Observational	ITNs	0.84^d^ (0.70 – 1.00)
Kenya [12]	Bondo, Greater Kisii, Kwale and Makueni	2004-06	Observational	ITNs	0.58^e^ (0.35 – 0.98)

a: Measured all-cause mortality in children aged 1-2 years comparing intervention to controls

b: Measured all-cause mortality in children aged 3-4 years comparing intervention to controls

c: Measured all-cause mortality in children aged 6-59 months comparing intervention to controls

d: Measured all-cause mortality in children aged 0-5 years comparing 1999 to 1997

e: Measured all-cause mortality in children aged 1-59 months comparing exposed to unexposed

CI: Confidence interval

### LiST model

Within each country, the LiST model at baseline uses estimates of the age structure of the population, fertility rates, <5 mortality rates, cause of death structure, and coverage estimates of key child survival interventions [13]. The model used in this analysis (Version 4.22) and accompanying documentation can be downloaded from http://www.jhsph.edu/dept/ih/IIP/list/. LiST estimates the number of cause-specific child deaths prevented each year, accounting for population growth, as the difference between the estimated deaths that occur with intervention scale-up and the estimated deaths that would have occurred without intervention scale-up beyond the coverage at a baseline year. The model estimates child deaths prevented by cause due to intervention scale-up as a function of three primary input parameters: 1) the number of child deaths by cause projected to occur in each year (including population growth parameters over time); 2) the protective effect on cause-specific mortality (protective effect = 1-relative risk*100) for each intervention being scaled-up; and 3) changes in population coverage of each intervention. Malaria is included in the model as a cause of death among children 1-59 months. For assessing the impact of vector control on all-cause child mortality, the LiST model requires the following 3 primary input parameters: 1) all-cause child mortality rate by age at baseline year; 2) the protective effect of vector control interventions on malaria mortality; and 3) yearly population coverage of vector control from baseline, which uses the indicator of the proportion of households owning at least1 ITN and/or receiving IRS within the past 12 months.

The LiST model uses a default 55% protective efficacy for estimating the impact of vector control interventions on preventing malaria deaths in children 1-59 months, based on a recent systematic review done specifically for LiST [14]. This estimate was derived from community-randomized controlled trials assessing the impact of ITNs on all-cause child mortality. The model uses ITN household possession as the intervention coverage indicator instead of ITN use by children because the trials from which 55% protective efficacy were derived all used intention-to-treat analyses, meaning the estimated effects (relative risk) were based on whether or not a child lived in a village with high coverage of household ITN possession. The estimates from these studies were therefore not based on whether children under the age of 5 slept under an ITN the previous night.

### Data used for comparison between LiST and study estimates

We began by using the standard demographic projection available for each country in LiST included in the analysis. We then used measured data for three key parameters in LiST for each comparison of modeled and measured estimates: household vector control coverage, proportion of post-neonatal mortality due to malaria, and baseline child mortality rate. The coverage of all other child survival interventions in LiST were held constant to ensure that only the effect of vector control on rates of all-cause child mortality was being modeled. The inputs used in LiST for each study comparison, as well as their sources, are detailed below (Table 2).

**Table 2:** Inputs used for the LiST model,

Study	Intervention Period	Baseline <5 mortality rate (5q0)	Baseline neonatal mortality rate	Proportion post-neonatal mortality due to malaria (%) [reference]	Intervention coverage baseline year	Intervention coverage year 1 (%)	Intervention coverage year 2 (%)
The Gambia [9]	1991-92	152^a^	41^b^	35 [15,16]	0%	80^c^	80^c^
Burkina Faso [10]	1994-96	254^d^	45^e^	39 [19]	0%	93	94
Tanzania [11]	1997-99	183^d^	44^e^	56^f^	10%	58	61
Kenya [12]	2004-06	109^d^	33^e^	29 [25]	0%	100	100

a: UNICEF country-specific mortality rates for the first year of the study [28]

b: Derived by subtracting child mortality 59q1 (in months) from child mortality 5q0

c: Estimated from study reporting that 80% of mosquito nets were treated in the intervention group [9]

d: Derived by adding the child mortality 59q1 (in months) to the neonatal mortality

e: Region-level estimates from DHS surveys [18,21,24]

f: Study specific estimate [22]

Three of the 4 studies measured all-cause child mortality as a rate of death (deaths per 1,000 person years), while the LiST model uses survival probabilities (probability of dying between birth and a child’s 5^th^ birthday [_5_q_0_ in years], or between 1 month and a child’s 5^th^ birthday [_59_q_1_ in months]). The fourth study measured all-cause child mortality as the probability of dying. Mortality rates per 1,000 person-years reported by the studies were therefore converted to survival probabilities using a life table analysis. Comparisons between LiST and the measured study estimates were done with unadjusted percent reductions in all-cause child mortality. Confidence intervals about mortality reduction estimates in the studies were calculated proportionally to the confidence intervals about the reported relative risk in each study.

The Gambia: The study was conducted in 1991-1992 and included 19,561 children in 104 villages matched on size and then randomly assigned to intervention or control [9]. The entomological inoculation rate (EIR), or number of infective bites per person per year, ranges from 1-10 in this area [9]. Villages were analyzed in pairs to account for correlated data. As the original study published the mortality rate among children 6-59 months, the all-cause child mortality rates 1-59 months were obtained from a Cochrane review on insecticide treated materials that included data on children 1-5 months from this study [4]. The neonatal mortality rate input into LiST was calculated by the difference between the <5 survival probability in 1991 (_5_q_0_) published by the Interagency Group on Child Mortality Estimation (http://www.childmortality.org/cmeMain.html) and the _59_q_1_ in months reported by the study. In the study publication, it was assumed that 80% of nets utilized in intervention villages were ITNs, thus the published coverage estimates of household possession of any mosquito net were multiplied by 80%. The baseline year coverage was set at 0%, as ITNs were unavailable in the control villages. The proportion of post-neonatal deaths due to malaria used in LiST was set to 34.8%, being the mean of 2 different studies measuring 35.0% in the upper river division of Gambia from 1989-1993 among children aged 1 to 59 months [15] and 34.6% along the southern bank of the Gambian river near the coast from 1988-1990 [16]. This second study from 1988-1990 included neonatal mortalities in its estimate of the proportion of child deaths due to malaria, and so the original figure of 25.3% was inflated by 26.9%, assuming that 26.9% of total child mortality occurred in the neonatal period in this area [15] and that malaria was not a significant cause of neonatal mortality.

Burkina Faso: The study was conducted from 1994-1996 and included 16,540 children in 168 villages aggregated to 16 randomized clusters [10]. The EIR averaged 300-500 per person in the area [17]. As the original study published the mortality rate among children 6-59 months, the all-cause 1-59 month child mortality rates were obtained from a Cochrane review on insecticide treated materials that included data on children 1-5 months from this study [4]. The neonatal mortality rate from the Platuea-Central region was used from the rural strata of the 1998-1999 DHS [18]. A demographic surveillance system in the Nouna Health District, which lies to the west of the study province, from 1997-1999 estimated the proportion of child deaths due to malaria to be 28.5% [19]; after adjustment to exclude neonatal deaths, assuming 26% of mortality occurring in the neonatal period [20], the proportion of deaths in children 1-59 months due to malaria was set to 38.5% in LiST. Yearly estimates of population ITC household coverage reported by the study were used.

Tanzania: The study was conducted in a rural area around Ifakara from 1997-1999. The EIR for this area ranges from 200-300 infective bites per person per year. The probability of a child dying before turning age 5 was measured both before and after the implementation of a social-marketing intervention to increase ITN coverage [11]. The neonatal mortality rate used in LiST was taken from the rural strata of the 1999 DHS [21]. The proportion of post-neonatal deaths due to malaria was set at 51.6%, the figure from ongoing surveillance from 1993-2001 measured in the rural areas of Morogoro Region [22]. Yearly estimates of population ITN household coverage reported by the study were used in LiST.

Kenya: The study was conducted from 2005-2007 and included 3,484 children in the analysis [12]. These children come from 4 different districts in Kenya, representing a range of different transmission settings that exist in Kenya [23]. All-cause child mortality for those exposed and unexposed to ITN use were published as the rate of death in children 1-59 months per 1,000 life years. The neonatal mortality rate for LiST was obtained from the rural strata of the 2008/2009 Demographic and Health Survey (DHS) [24]. The proportion of all deaths in children 1-59 months due to malaria was set at 28.8% obtained from the demographic surveillance system in Asembo-Gem (western Kenya) during 2002 [25]. Coverage of ITNs was set at 100%, as the study compared mortality rates among children that slept under an ITN and those that did not sleep under an ITN.

### Uncertainty about LiST estimates

The LiST software does not currently generate uncertainty or confidence intervals about modeled estimates. Instead, we performed a non-probabilistic sensitivity analysis by varying the primary parameters affecting malaria mortality to produce an uncertainty about the reduction of child mortality estimated by LiST. The protective efficacy of vector control was varied from 49% to 60% [14]. The <5 mortality rate, proportion of post-neonatal mortality due to malaria and the household vector control coverage were each varied by 10%; none of these studies provided confidence intervals or standard errors with their coverage estimates.

## Results

After converting all-cause child mortality rates (deaths per 1,000 person years) to survival probabilities of the same age range, the largest percent reduction in mortality from vector control intervention exposure was observed by the longitudinal descriptive study in Kenya, which reported a reduction of 24.6% [95% Confidence Interval (CI): 2.2% - 37.5%](Table 3). The ITN community-randomized controlled trial in The Gambia reported a 21.8% (95% CI: 4.7% - 35.1%) reduction in all-cause child mortality, while the ITC community-randomized controlled trial in Burkina Faso reported a 12.6% (95% CI: -7.9% - 27.9%) reduction. The Tanzanian study of socially-marketed ITNs reported the smallest reduction in all-cause child mortality of 7.9% (95% CI: 0.0% - 14.8%).

**Table 3:** Percent change in child mortality from observed study and LiST model predictions

Study [reference]	Intervention Period	Net intervention Coverage increase from baseline (%)	Child mortality reduction measured by study (%) (95% CI)	Child mortality reduction modeled by LiST (%) (Uncertainty Interval)	Relative difference between measured and modeled estimates (%)
The Gambia [9]	1991-92	80	21.8 (4.7 - 35.1)	17.1 (-3.1 – 33.3)	22
Burkina Faso [10]	1994-96	94	12.6 (-7.2 - 27.9)	18.7 (3.3 – 32.1)	33
Tanzania [11]	1997-99	51	7.9 (0.0 - 14.8)	12.1 (-1.2 – 24.5)	35
Kenya [12]	2004-06	100	24.6 (2.2 – 37.5)	18.4 (-4.6 – 36.8)	25

a: Study specific estimate

CI: Confidence interval

**Figure 1:**
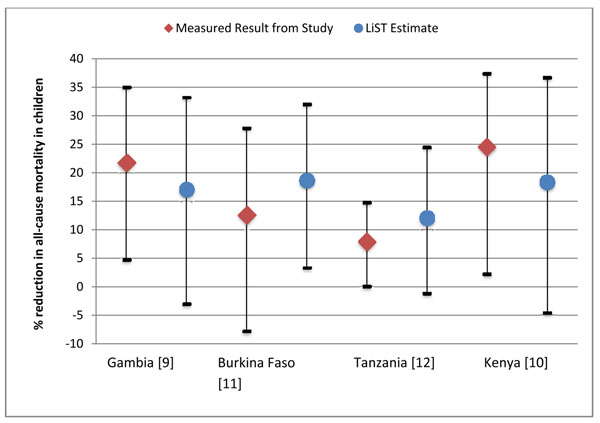
Vector control impact on child mortality with 95% confidence intervals reported by included studies compared to LiST estimations

After matching the baseline child mortality rate, proportion of post-neonatal mortality due to malaria and vector control intervention coverage in LiST to each study site to the extent possible, all four LiST-modeled estimates of the percent reduction in all-cause child mortality following vector control scale-up were within the 95% confidence intervals reported by the studies and quite close to the measured reductions (Figure 1). Similarly, the estimated reductions in mortality from the studies all fell within the uncertainty produced by the LiST model. The LiST-modeled estimates of the percent reductions were within ± 5 absolute percentage points of the measured reduction in The Gambia and Tanzania (relative difference between LiST estimates and measured data were 22% and 35%, respectively) (Table 3). The percent reductions in all-cause child mortality estimated by LiST were overestimated by 6.1 and 4.2 percentage points (33% and 35% relative to the measured estimates) in Burkina Faso and Tanzania respectively and underestimated by 4.7 and 6.2 percentage points (22% and 25% relative to the measured estimates) in the Gambia and Kenya respectively.

## Discussion and conclusions

The percent reductions in all-cause mortality as a result of vector control (ITNs and IRS) scale-up estimated with the LiST model were all within the published 95% confidence intervals from measured study data; all four studies had modeled estimates of child mortality reductions that came within 6.5 absolute percentage points of the measured changes. Furthermore, all of the study estimates fell within the uncertainty bounds of the reduction in child mortality calculated with the LiST model. The LiST model did not systematically under- or overestimate the impact of ITNs on all-cause child mortality, underestimating the impact for 2 studies while overestimating the impact for the other 2 studies. These results are consistent with LiST validation studies of other child survival interventions, suggesting that the 55% protective effect used in the model is a reasonable estimate of the potential impact of vector control on child mortality due to malaria, when matched with the population-level vector control indicator at the household level.

A potential reason that the LiST model estimates of reductions in all-cause child mortality differed slightly from measured reduction from studies is that only vector control was scaled-up in the LiST model; all other child survival interventions (e.g. exclusive breastfeeding and access to oral rehydration therapy) were held constant in the model due to a lack of data on their population coverage in the study sites. This is likely an inaccurate reflection what actually happened in the study sites, as even small changes in access to child survival interventions could have affected changes in measured rates of all-cause child mortality, which would not have been captured in the LiST estimates.

The LiST model estimate (17.1% reduction in 1-59 month all-cause child mortality) came closest to the measured results from the Gambia study (21.8% measured reduction in 1-59 month child mortality), underestimating the reduction by 4.7% (relative difference between measured and modeled estimates = 22%). A potential reason for the underestimation of LiST is that the proportion of post-neonatal mortality used in this analysis reflected only 2 of the 5 study areas, but did not include the study area with the highest measurements of malaria incidence. This area also saw the greatest reduction in child mortality. As such, the envelope of child malaria deaths that could potentially be prevented from ITN scale-up in the LiST model may have been underestimated, resulting in an underestimation of the impact of ITNs on child mortality.

The LiST model (18.4% reduction in 1-59 month all-cause child mortality) also underestimated the measured results from the Kenya study (24.6% measured reduction in 1-59 month child mortality, differing by 6.2 absolute percentage points [relative difference between measured and modeled estimates = 25%]). There are several possible reasons for this underestimation. First, the measured effect from the observational study in Kenya did not account for selection bias where children were self-selected into the ITN group, which may have caused an overestimate of the beneficial effect of ITNs [27]. Second, the Kenya study assessed the association of ITN use with child mortality (individual protection), while the LiST model uses ITN household possession. The ITN trials from which the 55% protective efficacy for LiST was derived measured the impact of whether or not a child lived in a village that had high household ITN possession, which is more equivalent to whether or not a child lives in a house protected by an ITN. Thus applying this ownership effect to the rate of utilization of ITNs would be expected to underestimate the population effect, as was actually seen.

The LiST model (12.1% reduction in all-cause <5 child mortality) overestimated the measured results from the Ifakara Tanzania study (7.9% measured reduction in <5 mortality), differing by 4.1 absolute percentage points (relative difference between measured and modeled estimates = 35%). A potential reason for the overestimation of LiST to the measured estimate is that the LiST input parameter for the proportion of post-neonatal mortality due to malaria (56%) was obtained from a single unpublished study in the site, and may be an overestimation of the true proportion of all child deaths due to malaria. As such, the envelope of child malaria deaths that could potentially be prevented from ITN scale-up in the LiST model may have been overestimated, resulting in an overestimation of the impact of ITNs on child mortality.

LiST also overestimated the percent reduction in 1-59 month all-cause child mortality (18.7% reduction) compared to the Burkina Faso ITC trial (relative difference between measured and modeled estimates = 35%), which showed a 12.6% reduction in 1-59 month child mortality among children in ITC intervention clusters. A potential reason for this difference is that ITCs may be less effective than ITNs, which form the basis of the 55% protective efficacy used in LiST.

Other attempts to validate mortality reductions estimated by LiST over longer time periods and incorporating multiple child survival interventions have shown LiST to perform reasonably well against measured data on child mortality in various settings. One such study attempted to model the reduction in mortality due to the implementation of UNICEF’s Accelerated Child Survival and Development (ACSD) program in Ghana and Mali from 2001 to 2005, matching mortality rates and intervention coverage measured through household surveys [8]. LiST-estimated all-cause child mortality rates fell inside the confidence interval of measured mortality rates in the Ghana comparison, but outside the confidence interval of measured mortality rates in the Mali comparison. Another study assessed LiST’s ability to model neonatal mortality reductions due to community-based interventions from 4 published studies in Bangladesh, India and Pakistan, from 1993-2007 [7]. LiST-estimated neonatal mortality rates fell within the 95% confidence intervals of the measured reduction in neonatal mortality rate for 3 of the 4 studies.

These results show the LiST model to perform adequately at estimating the effect of vector control scale-up on child mortality when compared against measured data across a range of malaria transmission patterns and intensities. It would appear to be useful in assessing likely impact in populations in high transmission areas in sub-Saharan Africa and in multi-country or regional assessments and for multi-year examination of progress. With few input parameters, the LiST model is relatively simple. However, simplicity is preferred if the model performs well, which has been shown with this comparison of 4 studies that achieved rapid scale-up. More study comparisons should be performed to assess how well LiST estimates reductions in child mortality when vector control intervention scale-up does not occur as rapidly or occurs in combination with other health interventions.

There is increasing pressure for malaria control programs to demonstrate the impact of malaria intervention scale-up on saving lives. Unfortunately, child mortality data from vital registration remain sparse across most of Africa, precluding their use for measuring changes in malaria mortality at national levels. The LiST model is a useful tool and can be used to estimate the potential impact of different scenarios of scaling-up malaria control interventions, especially where resources are limited. LiST modeled estimates will undoubtedly be most useful when coupled with population-level data, especially from nationally-representative surveys, showing intervention coverage has increased while all-cause and malaria-specific child mortality have decreased.

## Competing interests

The authors declare that they have no competing interests.

## Authors’ contributions

DL and TE designed the study and performed all analyses. IKF advised on the LiST analyses. DL wrote the first draft. DL, IKF and TE were all involved in rewriting the article and summarizing the conclusions.
